# Exploring the mental well-being of Ekurhuleni primary healthcare clinic managers during COVID-19

**DOI:** 10.4102/hsag.v30i0.2786

**Published:** 2025-01-17

**Authors:** Sanele E. Nene, Siyabulela N. Wopula

**Affiliations:** 1 Department of Nursing, Faculty of Health Sciences, University of Johannesburg, Johannesburg, South Africa

**Keywords:** mental well-being, primary healthcare, clinic managers, stress, COVID-19

## Abstract

**Background:**

The coronavirus disease 2019 (COVID-19) pandemic extensively disrupted the management dynamics and stretched the mental well-being of Ekurhuleni primary healthcare clinic managers. Their workload was increased, and they also had to deal with the grief of losing colleagues, family members and patients in large numbers as a result of the pandemic.

**Aim:**

This study sought to explore and describe the mental well-being of Ekurhuleni primary healthcare clinic managers during the COVID-19 pandemic.

**Setting:**

This study was conducted in the primary healthcare clinics of Ekurhuleni region in Gauteng province.

**Methods:**

A qualitative, exploratory, descriptive and contextual research design was adopted in this study. The sample size comprised 14 clinic managers. Purposive sampling was used to select the participants, and in-depth semi-structured individual interviews were conducted to collect data. The descriptive thematic analysis method was used to analyse data.

**Results:**

Four themes emerged from the study: (1) unbearable level of stress because of the new management dynamics, (2) fear and anxiety because of a sudden escalating death rate, (3) unresponsive employee wellness programmes and (4) lack of support from senior management.

**Conclusion:**

The senior management of primary healthcare clinics should safeguard the mental well-being of clinic managers during the pandemic by giving support and ensuring that the existing employee wellness system is responsive.

**Contribution:**

This study revealed that a strong support from senior management and responsive employee wellness programmes can strengthen the mental health of the primary healthcare clinic managers. Thus, this enables them to be future ready for possible pandemics like COVID-19.

## Introduction

### Background

Coronavirus disease 2019 (COVID-19) is a severe acute respiratory syndrome characterised by fever, coughing and shortness of breath. It was first detected in Wuhan, China, in December 2020, and the World Health Organization declared it a pandemic on 11 March 2020 (Lipsitch, Swerdlow & Finelli 2020). The primary healthcare (PHC) clinics in South Africa where this study was conducted experienced significant changes from March 2020, when the pandemic became an uncomfortable reality in healthcare clinics, influencing management dynamics (Wopula, Nene & Nkosi 2022). Hospital admission numbers increased, and most patients were admitted to intensive care units needing ventilation (Moosa 2020).

The emergence of COVID-19 devastated the South African healthcare system, which was already overwhelmed by the country’s burden of disease and resource shortage, thus adversely affected the mental well-being of PHC clinic managers (World Health Organization 2021). The mental state of these managers was affected by the significantly increasing mortality rate of patients, nurses, their family members and fear that even themselves might die from the pandemic especially considering that some of them were living with comorbidities (Dohrn et al. 2022). Primary healthcare clinic managers and nurses experienced practice changes, fear for themselves and their families and faced moral distress because of their inability to provide optimal care because of imposed restrictions as they were expected to minimise contact with their patients during the pandemic.

Throughout this time, it was imperative for clinic managers to be resilient and provide the needed support for nurses and other staff in PHC clinics. Their leadership roles entailed leading the self, leading others, and leading the organisation, which took a toll on them as they soon felt overwhelmed (Wopula et al. 2022). Primary healthcare clinic managers had to cater to the nursing staff’s needs and balance these with the duty to provide quality nursing care, focus on data and ensure human and financial resource management, which were significant challenges during the pandemic (Nene 2022). Altuntas, Dereli and Kaya (2020) state that effective staff support fosters a positive workplace culture that results in increased patient satisfaction and staff well-being. With the exhaustion of resources, operational managers needed to use clinical nurses’ decision-making skills to preserve trusting relationships between themselves, nursing staff and patients. Moreover, inconsistent information and social media’s sensationalisation of the pandemic added to the crisis, and operational managers realised the increased need for clinical nurses (Wopula et al. 2022).

The efficient management of healthcare providers and healthcare systems during the initial stages of COVID-19 demanded clinic managers use their basic skills and knowledge of nursing management as the healthcare system shifted and required a great deal of teamwork and preparation in this time of uncertainty (Roe et al. 2022). Primary healthcare clinic managers were forced to return to the basics of practicing fundamental nursing management dynamics as the pandemic necessitated prompt planning and swift escalation of nursing resources.

Primary healthcare is a pivotal component of the South African healthcare system as it affords low and middle-income earners (84% of the population) equitable access to health care, mitigating the burden of diseases and death rate in the country. McKenzie et al. (2017) elicit that PHC clinic managers oversee the overall operational activities in the PHC clinics. The operational managers also ensure that effective management processes and systems are in place in these settings (The Republic of South Africa National Department of Health Strategic Plan 2020/21–2024/25). Primary healthcare clinic managers are expected to be competent in operational management, quality improvement, information management, service delivery, resource management, infection prevention and control, leadership and cooperative governance (Jooste 2017; Wopula et al. 2022).

### Problem statement

The author observed that the PHC clinics headcount tremendously increased during the COVID-19 pandemic as more patients required medical attention of which most of it was emergency treatment because of the pandemic. More staff contracted the virus and were not fit for work meaning that clinic managers had to disregard their managerial roles and assist with clinical activities. The workload increased while staff compliment was dropping (Huang et al. 2020). Moreover, these managers were traumatised by the increasing number of patients, colleagues and their loved ones who were succumbing to the pandemic. This had a negative influence on the mental well-being of the operational managers as they had to deal with uncertainty, great confusion and the most difficult experience in the history of their nursing management (Wopula et al. 2022). The World Health Organization (2021) reported that COVID-19 was not a common pandemic, but it was a global healthcare crisis which left the healthcare workers and operational managers mental state disrupted. Greenberg et al. (2020) posited that the mental well-being of operational managers was at stake as they were expected to perform miracles during the pandemic; they had to stretch themselves using scanty resources while numbers of patients seeking medical attention tripled. Aloweni et al. (2022) suggested that it should be interesting to explore the extent to which COVID-19 troubled the mental well-being of the operational managers in PHC clinics. This problem resulted in the following research question: *How was the mental well-being of Ekurhuleni primary healthcare clinic managers during COVID-19?*

### Purpose of the study

The purpose of the study was to explore and describe the mental well-being of Ekurhuleni PHC clinic managers during COVID-19.

### Setting

The study was conducted in Gauteng province, in the PHC clinics of Ekurhuleni district, East Region which comprise 28 clinics providing comprehensive PHC services, operating 5 days a week for 8 h, while some of them are also operating on Saturdays from 07:30 to 14:00.

## Research methods and design

The study used a qualitative, exploratory, descriptive and contextual design, which assisted the researcher in gaining an in-depth understanding of PHC clinic managers’ mental well-being during the COVID-19 pandemic. The author deemed this design relevant for the study as it delved on gaining an understanding of the mental well-being of the clinic managers during COVID-19 pandemic through naturalistic observations and exploration of reality (Polit & Beck 2018).

The following research methods were adopted in this study.

### Population and sample

The population for this study consisted of all PHC clinic managers in the Ekurhuleni health district, totalling to 28 operational managers in total. The sample frame of this study was 14 clinic managers from the Ekurhuleni east region who met the inclusion criteria. Purposive sampling was chosen for this study, and it assisted the researcher in choosing participants with in-depth knowledge about the phenomenon of interest to gain comprehensive information from them. The sample size was not predetermined but depended on data saturation. Data saturation was attained by the seventh interview when no new information was received from participants, but two more interviews were conducted to confirm the data saturation.

#### Inclusion criteria

The inclusion criteria of this study were PHC clinic managers with two or more years’ work experience in a clinic management permanent post in the Ekurhuleni health district, who were in this position during the COVID-19 pandemic, and who agreed to participate in the study.

#### Exclusion criteria

Primary healthcare clinic managers who had less than 2 years work experience, not in this position during COVID-19 pandemic, and those who were on leave during data collection were excluded from this study.

### Data collection

After obtaining the permission to conduct the study, the author arranged a recruitment meeting with all the prospective participants in one of clinic managers regular review meeting. The research title, purpose, methods, central question, risks and benefits were discussed in this meeting, and all questions were clarified. Participants who demonstrated interest to participate in the study signed the participation informed consent forms. Data were collected using in-depth semi-structured individual interviews by the researcher who had no personal relationship with any of the participants. The interviews were scheduled and conducted at the participants’ convenience, either at the facilities’ boardrooms or clinic managers’ offices, on the date and time suiting their work schedule. Masks and sanitisers were readily available to ensure COVID-19 protocols were adhered to such as social distancing, and water was available for participants. All participants chose to be interviewed in English, and each interview lasted approximately 45–60 min. The participants were asked one central question: ‘How was your mental well-being during the COVID-19 pandemic?’ followed by probing to seek clarity on the participants’ responses. Moreover, field notes were recorded during the interviews to capture participants’ non-verbal expressions.

### Data analysis

Data were analysed using Giorgi’s five-step descriptive thematic analysis method presented in Table 1 (Polit & Beck 2018).

**TABLE 1:** Five steps of thematic data analysis.

Step	Method
Step 1	In this step, all the data from the audio-recording and field notes were transcribed verbatim.
Step 2	The researcher then made a brief outline of the information that was transcribed to draw meaning from the words participants used in the interview’s entirety.
Step 3	The researcher became immersed in the transcripts to categorise units of meaning.
Step 4	In this step, the researcher created a general description of the phenomenon under study within the operational managers’ context as explained by them.
Step 5	Data analysis required themes and categories to be grouped together. An independent coder was sourced to verify the themes and categories and reach a consensus about the findings with the researcher.

*Source*: Adapted from Polit, D.F. & Beck, C.T., 2018, *Nursing research: Generating and assessing evidence for nursing practice*, Lippincott Williams & Wilkins, Philadelphia, PA

## Trustworthiness

To ensure trustworthiness in this study, the researcher used Lincoln and Guba’s (2016) quality criteria framework, namely credibility, transferability, dependability and confirmability. The researcher ensured prolonged engagement with the participants in the field to extensively understand their context and triangulated data to establish credibility (Holloway & Galvin 2023). Triangulation was ensured by collecting data using in-depth individual interviews and recording field notes, and bracketing was pre-empted through regular supervision feedback sessions during this process. To ensure the findings’ transferability, the researcher extensively discussed the research methods and the context of the study. Dependability was maintained by holding regular correction and feedback meetings with the study’s supervisors. Confirmability was attained during back-and-forth meetings with the independent coder, researcher and supervisors to discuss the findings until consensus was reached (Lincoln & Guba 2016, Wopula et al. 2022). Moreover, peer review was explicitly executed throughout the research process.

### Ethical considerations

The following ethical principles were upheld throughout the study: autonomy, justice, non-maleficence and beneficence (Dhai & McQuoid-Mason 2020). The ethical clearance was obtained from the University of Johannesburg Research Ethics Committee and Ekurhuleni health district (Clearance Number REC-1264-2021). All the participants granted the permission to participate in the study in writing, and were advised that they can withdraw from participating anytime. Confidentiality was maintained by allocating codes to data to protect the personal details of the participants. There was no harm envisaged for participating in the study and the findings of the study were presented to the participants.

## Results

The study’s findings emanated from purposively selected clinic managers’ in-depth, individual interviews. These managers were from PHC clinics in the Ekurhuleni health district and had between 2 and 35 years’ experience in their position. Five female participants between 38 and 61 years, and four male participants between 42 and 44 years, participated in the study. All the participants were Africans and held a Diploma in General Nursing, Midwifery, Psychiatry and Community Nursing and a post-basic Diploma in Clinical Nursing Science, Health Assessment, Treatment and Care (Regulation 48). Moreover, eight participants held post-basic B.Cur. Ed Admin Occupational Health degrees, and one participant had a Master’s degree in Nursing Management. Table 2 illustrates the demographic information of the participants.

**TABLE 2:** Summary of participants’ demographic information.

Participant	Ethnicity	Age (years)	Qualification	Experience	Gender
P1	African	43	General Nurses, Midwifery, psychiatry and Community Nursing, Diploma in Clinical Nursing Science, Health Assessment, Treatment and Care (R48), Occupational Health Nursing Science, Education and Administration.	10 years	Male
P2	African	61	General Nurses, Midwifery, psychiatry and Community Nursing, Diploma in Nursing Management, Diploma in Clinical Nursing Science, Health Assessment, Treatment and Care (R48).	35 years	Female
P3	African	59	General Nurses, Midwifery, psychiatry and Community Nursing, B.Cur Nursing Science, Diploma in Clinical Nursing Science, Health Assessment, Treatment and Care (R48).	26 years	Female
P4	African	42	General Nurses, Midwifery, psychiatry and Community Nursing, Diploma in Nursing Management, Diploma in Clinical Nursing Science, Health Assessment, Treatment and Care (R48), Master’s in Nursing Management.	8 years	Male
P5	African	52	General Nurses, Midwifery, psychiatry and Community Nursing, Diploma in Nursing Management, Diploma in Clinical Nursing Science, Health, Assessment, Treatment and Care (R48), B.Cur Ed Et Admin Occupational Health Nursing Science.	8 years	Female
P6	African	42	General Nurses, Midwifery, psychiatry, and Community Nursing and Diploma in Clinical Nursing Science, Health Assessment, Treatment and Care (R48), Occupational Health Nursing Science, Education and Administration.	2 years	Male
P7	African	38	General Nurses, Midwifery, psychiatry and Community Nursing, Diploma in Nursing Management, Diploma in Clinical	8 years	Female
P8	African	44	General Nurses, Midwifery, psychiatry and Community Nursing, Diploma in Nursing Management, Diploma in Clinical Nursing Science, Health Assessment, Treatment and Care (R48), B.Cur Ed Et admin Occupational Health Nursing.	4 years	Male
P9	African	59	General Nurses, Midwifery, psychiatry and Community Nursing, Diploma in Clinical Nursing Science, Health Assessment, Treatment and Care (R48), Education and Administration.	12 years	Female

Four themes emerged as findings of this study and are presented next.

### Unbearable level of stress because of the new management dynamics

The study’s findings revealed that participants were emotional and physically distressed because of scarce resources, the constant threat of being infected with the COVID-19 virus, other occupational hazards, prolonged working hours, workplace disruptions, poor sleep patterns and inability to balance work and social life. All these factors exacerbated their physical and psychological exhaustion, anxiety, stress and burnout, as confirmed in the following quotations:

‘I had little or no time for myself as a clinic manager, when I come to the staff, I had to act strong and portray everything is going to be alright attitude, it was very, it was very difficult. I felt at some point I was depressed with all the burden I was carrying in my shoulders; I was very emotionally drained and really couldn’t take it [*laughing*].’ (P7, female, 38 years)

‘To go and test people in their workplaces even at out-reach campaigns of which at the clinic you were like once or twice per week of which the work that is supposed to be done at the clinic seriously affected me, yes to tell you the truth the stress level I couldn’t take it [*looking up at the ceiling holding her head, emotional*].’ (P8, male, 44 years)

Another participant added:

‘With us, remember the management aspect the is an issue and now additionally with the COVID-19 you have to oversee everything that comes with COVID-19 demands – there is shortage of staff and now you have to put your management duties aside and do the clinical work and then continue with your management duties, physically I was not just drained but I was physically stressed too, it was too much.’ (P1, male, 43 years)

### Fear and anxiety because of a sudden escalating death rate

The following verbatim quotations are directly linked to this theme, reflecting participants’ fear and anxiety because of a sudden escalating death rate attributed to the pandemic:

‘[*Taking a deep breath*] So mentally, the human resource, you know the staff were panicking, fearing for their lives as the death rate increased because of the pandemic.’ (P2, female, 61 years)

‘And then fearing for their families, so absenteeism went high as family members succumbed to the COVID-19 virus.’ (P3, female, 59 years)

Other participants mentioned the following:

‘That ended up causing a lot of staff members eh … being disgruntled, that is number one, that we are overloaded, and number two, with the fear of them contracting the disease itself because of overloading of patients at one time in the clinic.’ (P7, female, 38 years)

‘So mentally the human resource, you know the staff were panicking, fearing for their lives as the death rate increased because of the pandemic … And then fearing for their families, so absenteeism went high [*getting emotional*].’ (P5, female, 52 years)

### Unresponsive employee wellness programmes

Participants reported that the employee wellness programme was not responsive during the pandemic. The following verbatim quotations support this view:

‘The employer did send us emails again about EAP that was going to be available to us during these hard times … But believe me when you call those people, they were never available, that phones would ring alone no one will answer the phone, so there was no support I’m telling you, there was no external support I will say so [*looking worried*].’ (P6, female, 42 years)

‘EAP department to come to party they must show up, because you will phone them, then they will be telling you they are working from home they can’t take calls now, phone this person, phone that person they just need to attend the staff, debrief the staff.’ (P5, female, 52 years)

‘We needed EAP most during this time because this whole thing was overwhelming for all of us, but our EAP failed us, we couldn’t get hold of them and they were not returning our calls, for some reasons I thought that they were on leave or something [*taking a deep breath*].’ (P1, male, 43 years)

### Lack of support from senior management

The participants also mentioned that there was a lack of support from the senior management during this difficult time. The following quotations are confirming this statement:

‘With the poor support from our management because really I didn’t feel any support from our senior management except the emails that they were sending [*silence … silence*], there was nothing really it was just us working harder, stretching further.’ (P2, female, 61 years)

‘Look there was no support from the management, you would literally hardly hear from them.’ (P6, female, 42 years)

‘To be honest with you I was very disappointed on our senior management, no support in such a difficult time, not even showing up as they used to do on normal days [*looking angry and using both hands to explain*].’ (P8, male, 44 years)

## Discussion

### Unbearable level of stress because of the new management dynamics

The participants reported that they were under an unbearable level of stress because of the new management dynamics caused by COVID-19. Shechter et al. (2020) agree that the pandemic caused an unbearable level of stress for clinic managers as their workload increased and they were expected to also execute clinical tasks. On the same note, Giorgi et al. (2018) alluded unbearable work-related stress experienced by the managers during this difficult time contributed to their emotional turmoil and burnout, and this was exacerbated by adverse working conditions.

Primary healthcare clinic managers were bound to experience emotional and mental breakdowns during the COVID-19 pandemic, because how can one lead effectively with limited resources, while overloaded with work (Dohrn et al. 2022). The instant outpouring of COVID-19 cases, accompanied by ambiguity in treatment protocols, increased the PHC clinic managers’ stress levels, and frustrations (Azoulay et al. 2020). The new dynamics of PHC clinic managers’ expanded service delivery roles during the pandemic and yielded emotional and physical stress. Based on collected data, there was an emotional turmoil for PHC clinic managers which negatively affected the quality of care received by patients as the service providers were psychologically and physically compromised.

### Fear and anxiety because of a sudden escalating death rate

The participants were scared and anxious as more individuals were losing their lives from COVID-19. Hence, the World Health Organization (2021) reported that COVID-19 resulted in escalated infections, ill health and deaths among healthcare providers and estimated that about 80 000 healthcare workers died from COVID-19 globally in the period from January 2020 to May 2021. The Republic of South Africa National Institute for Communicable Disease (2021) reported that South Africa felt the devastating effects of COVID-19 during the pandemic’s second wave in December 2020, with a cumulative total death toll of 24 907 by 21 December 2020.

Walton, Murray and Christian (2020) posit that healthcare workers are trained to care families losing their loved ones, but the deaths of the pandemics frightened them as they were happening at a high scale, affecting everyone. Shechter et al. (2020) agreed that clinic managers are amongst the healthcare workers who took an oath to save lives; this time they had to do this while in fear and anxious about whether they will survive the next day or not. Lam et al. (2019) concur that the COVID-19 deaths were shocking to the public, even more on the healthcare workers who were at the forefront of the pandemic. Fear and anxiety of the clinic managers exacerbated their physical and psychological exhaustion, considering that they had to work extended hours as the pandemic was taking its toll (Raudenská et al. 2019).

Despite having a response plan, the governments across the globe initially struggled to curb the escalating death rate. The pandemic raged through society, and healthcare workers remained in the line of duty, even though they felt stressed and anxious about contracting the virus and infecting their families (Huang et al. 2020). In agreement with this is Kang et al. (2018) as they stated that the PHC clinic managers and nurses had to face this dangerous situation, they were steadfast in their roles and commitment of healthcare provision. Based on the responses of the participants in this theme, it became evident to the researcher that PHC clinic managers and nurses were contently anxious and fearful of the COVID-19 pandemic. It adversely affected their mental well-being as many healthcare providers became ill, and some lost their lives to the pandemic.

### Unresponsive employee wellness programmes

According to the participants of this study, the existing employee wellness programmes were unresponsive. World Health Organization (2021) agreed that based on the nature and magnitude of the pandemic, almost every healthcare organisation required a strong and responsive Employee Assistance Programme (EAP). Wopula et al. (2022) affirm that the clinic managers yearned for debriefing sessions and access to EAP. Melnyk et al. (2018) reveal that the pandemic exposed the quality of EAPs available in the healthcare sector, as most of them were overwhelmed and unresponsive. The EAPs were not adequately equipped to deal with the pandemic; hence, their resources were depleted or not available at all during such a needy time (Mo et al. 2020).

Nurses and nurse managers reported severe anxiety and depression during the pandemic (Melnyk et al. 2018). Hence, in that same vein, Walton et al. (2020) concluded that the mass traumatic encounters brought about by COVID-19 affected clinic managers’ and nurses’ mental well-being. Melnyk et al. (2018), Shechter et al. (2020) and Walton et al. (2020) reported that EAP companies should be held accountable for being unresponsive during COVID-19; consequently, this severely affected the mental well-being of the clinic managers as they had mass traumatic encounters. Information from the Ekurhuleni database concurred that nurses and clinic managers were overwhelmed and seeking assistance through employee well-being programmes which were unresponsive (Dohrn et al. 2022). However, not all individuals could be accommodated because of the increased demand. On the other hand, the City of Ekurhuleni Employee Wellbeing Department (2021) reported that because of the increasing demand in employee wellness, not all individuals were accommodated, and this resulted in some individuals sharing that the EAPs within their organisations were ineffective.

### Lack of support from senior management

Lack of support from senior management emanated as the final theme of this study. Nene, Ally and Nkosi (2020) agree that fulfilling operational obligations for clinic managers without support from the senior managers is a difficult and frustrating task, compromising the quality of service delivered. Liu et al. (2020) and White (2021) added that during COVID-19 the clinic managers had to manage the increasing patient complaints and dissatisfaction, while attempting to swiftly embrace their new normal by restructuring the PHC clinics without the support from the senior management.

Nene (2024) concurs that most senior managers failed clinic managers as they expected them to effectively navigate the pandemic unsupported, disempowered, ill-equipped and working under devastating working conditions. Andrews, Tierney and Seers (2020) attest that when senior management support diminishes, clinic managers should adopt self-caring and self-compassionate strategies to uphold their wellbeing especially during difficult times of such a pandemic. Serapelwane and Manyedi (2020) annotated that lack of support from senior management hit hard on the clinic managers as it become difficult for them to perform their management obligations such as detecting challenges in advance and taking actions. Gomes (2022) postulated that it was unhuman of the senior management not to support clinic managers during the pandemic as they needed their support most. The participants’ perceptions of unresponsive employee wellness programmes and lack of support from senior management during the pandemic indicated much of what PHC clinic managers went through could have been avoided, or its effects could have been minimised. They emphasised the need for responsive EAPs and a strong support from senior management during the pandemics of such a calibre.

### Recommendations

All nurses and clinic managers should be referred to the EAP team for debriefing to alleviate stress, fear and anxiety. Senior management must double their efforts and support nurses and clinic managers to alleviate their stress and anxiety for them to be able to make ethical decisions and increase zeal in performing their duties during difficult times. The existing employee wellness programmes should be contextualised and be adaptable to respond in times of pandemics and to ensure a healthy mental wellbeing of PHC clinics managers.

### Limitations

This study’s primary focus was on Ekurhuleni PHC clinic managers’ mental well-being during the COVID-19 pandemic. Therefore, the scope and overview of the study were limited to these individuals. All other categories or carders in the Ekurhuleni PHC clinics such as medical practitioners, nurses, administrators, cleaning personnel and other supporting personnel did not form part of the study.

## Conclusion

This study revealed that the mental well-being of the PHC clinic managers was compromised by the altered management dynamics caused by COVID-19 pandemic, and it led to unbearable level of stress, fear and anxiety. The existing wellness EAPs were unresponsive, and there was a lack of support from the senior management.
